# A Rare Afferent Loop Syndrome Case in Adulthood Following Liver Transplantation in Neonatal Hemochromatosis

**DOI:** 10.1016/j.gastha.2023.11.013

**Published:** 2023-12-01

**Authors:** Meghan R. Mansour, Thomas D. Meram, Steven A. Kessler, Ali Khreisat, Adam Wernette, Justin K. Skrzynski

**Affiliations:** 1 Oakland University William Beaumont School of Medicine, Rochester Hills, Michigan; 2 Department of Internal Medicine, Corewell Health William Beaumont University Hospital, Royal Oak, Michigan

**Keywords:** Afferent Loop Syndrome, Afferent Limb Syndrome, Neonatal Hemochromatosis, Anastomotic Stricture, Hepatobiliary Complications

## Abstract

Afferent loop syndrome, sometimes referred to as afferent limb syndrome, is an infrequent mechanical complication frequently observed following foregut surgeries involving the connection of the stomach or esophagus to the jejunum. This condition is commonly found in individuals who have undergone Billroth II reconstruction following a partial gastrectomy. Here, we present the first documented case of afferent loop syndrome in a patient with a medical history involving a liver transplant due to neonatal hemochromatosis.

## Introduction

Afferent loop syndrome (ALS), also known as afferent limb syndrome, is a rare mechanical complication commonly observed after foregut procedures that entail the anastomosis of the stomach or esophagus with the jejunum.^1^ This condition was initially documented in postgastrectomy patients presenting with bilious vomiting,^2^^,^^3^ predominantly found in individuals with Billroth II reconstruction subsequent to partial gastrectomy.^4^ We present the first documented case, to our knowledge, of ALS in a patient with a history of liver transplant secondary to neonatal hemochromatosis.

## Case Report

Our patient is a 29-year-old female with a history of neonatal hemochromatosis status post-liver transplantation at 8 weeks of age; complicated by recurrent cholangitis, bile duct obstructions, and multiple percutaneous transhepatic catheter placements for drainage and biliary cast development. She was transferred to our care following an enteroscopy performed at an external medical facility, which identified an anastomotic stricture between the bile duct and jejunum with associated choledocholithiasis. Her vital signs were stable upon presentation, and pertinent labs revealed: aspartate aminotransferase 130 IU/L, alanine aminotransferase 264 IU/L, bilirubin 2 mg/dL, and alkaline phosphatase 193 IU/L. The patient reported a history of chronic abdominal pain and was receiving an opioid-based regimen under the management of palliative care. Two months prior to admission, the patient had a magnetic resonance cholangiopancreatography performed, which identified several dilated biliary radicles within the right aspect of the liver, concerning a possible stricture. Additionally, a recent endoscopic ultrasound with biopsy was negative for liver transplant rejection.

During this hospitalization, an endoscopic retrograde cholangiopancreatography was conducted, revealing substantial looping in the jejunal afferent limb, which required abdominal pressure to advance the scope to reach the hepaticojejunostomy. Endoscopic retrograde cholangiopancreatography also revealed a moderately benign-appearing, intrinsic stenosis in the jejunum downstream from the hepaticojejunostomy with a significantly dilated lumen around the hepaticojejunostomy, raising concern for a possible afferent limb syndrome. The right intrahepatic branches were moderately and segmentally dilated secondary to a stricture. A 7-mm balloon dilator was successfully used to mechanically dilate the right main hepatic duct and the intrahepatic branches; additionally, sludge was swept from the duct with a 10-mm balloon in these ducts. The procedure was uncomplicated and well-tolerated by the patient. She experienced only mild abdominal discomfort and nausea after the procedure, both of which resolved during her recovery.

Postprocedure magnetic resonance cholangiopancreatography showed no evidence of residual choledocholithiasis, and the jejunal afferent segment was not in close proximity to the stomach or duodenum. Mildly diffuse dilation of intrahepatic ducts, containing both bile and gas, was observed within the anterior liver along its periphery. Figure demonstrates a dilated afferent jejunal segment in both **a)** coronal view and **b)** transverse view computed tomography. These findings aligned with the suspected diagnosis of afferent limb syndrome. Her chronic abdominal pain also supported this diagnosis. Options for surgical intervention vs endoscopic stenting were considered at this point. The patient received guidance to schedule follow-up appointments with both transplant surgery and gastroenterology outpatient services to determine the subsequent course of action. However, as of writing this case, the patient has not pursued any further consultations with outpatient providers.

**Figure:**
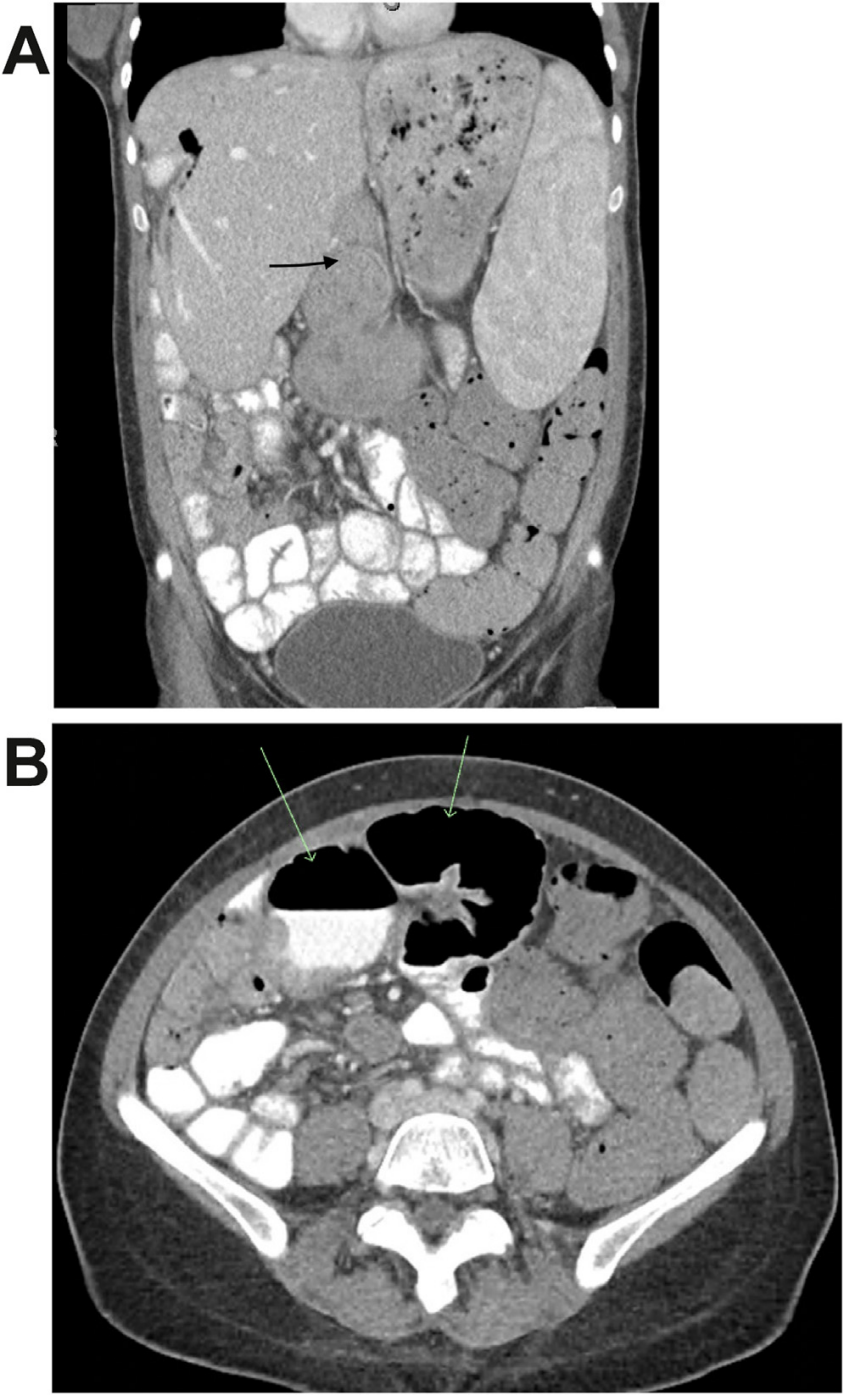
(A) Coronal view CT showing a dilated afferent jejunal segment (B), transverse view CT with green arrows showing dilated afferent jejunal segment. CT, computed tomography.

## Discussion

While the majority of ALS cases emerge in the initial postoperative phase, there's still an increased risk even for those with remote surgical histories, as in our patient’s case.^5^ ALS can arise from any obstructive process that affects the afferent limb of the anastomosis or at the distal anastomosis site. These obstructions can be either inside or outside the intestinal lumen, encompassing factors such as hernias, adhesions, scar tissue, gastric bezoars, biliary stones, and cancer. The severity of the obstruction, whether it's complete or partial, and the degree of bowel distention and blockage in the pancreaticobiliary tree dictate the acuity of the disease and the patient’s presentation.^4^ Stagnation of bile and pancreatic secretions can heighten susceptibility of patients with ALS to ascending cholangitis and pancreatitis. Moreover, the afferent limb presents an increased risk of small intestinal bacterial overgrowth, possibly leading to a condition like blind loop syndrome.^4^

Patients with ALS may present acutely or chronically, depending on the etiology and degree of obstruction. The wide range of presenting symptoms includes abdominal pain, nausea, vomiting, a palpable abdominal mass, jaundice, and features of malabsorption. Interestingly, our patient presented with a history of seemingly idiopathic chronic abdominal pain, unrelieved by a long-term opioid-based regimen. Diagnosing ALS demands a strong clinical suspicion, particularly as seen in this instance, which is the first documented case of post-liver transplant ALS in a patient with hemochromatosis.

The management of ALS in patients depends on symptom severity and the underlying cause. Surgery is typically the definitive approach for those with benign etiologies, such as postsurgery strictures. However, minimally invasive procedures may be more cost-effective and better tolerated, especially for individuals under palliative care. Additionally, the diagnosis and management of ALS could be limited by available clinical resources, including the availability of gastroenterology practitioners with the necessary advanced endoscopic training. Nonetheless, it is important to encourage all gastroenterologists to include ALS as part of their differential diagnoses for patients presenting with both classic and ambiguous symptoms. In the case of our patient, her transfer to our care was prompted by her need for specialized treatment.
